# Photo-Regeneration of Zeolite-Based Volatile Organic Compound Filters Enabled by TiO_2_ Photocatalyst

**DOI:** 10.3390/nano12172959

**Published:** 2022-08-26

**Authors:** Taegyu Kim, Kunsang Yoo, Myung-Gil Kim, Yong-Hoon Kim

**Affiliations:** School of Advanced Materials Science and Engineering, Sungkyunkwan University, Suwon 16419, Korea

**Keywords:** volatile organic compounds, photo-regeneration, ultraviolet, zeolite, titanium dioxide

## Abstract

Indoor air filtration received significant attention owing to the growing threat to the environment and human health caused by air pollutants such as volatile organic compound (VOC) gases. However, owing to the limited adsorption capacity of VOC adsorbents, such as activated carbon, a rapid breakthrough can occur, reducing the service life of the filter. Therefore, TiO_2_-coated zeolite (TiO_2_/zeolite) was utilized as a photo-regenerative VOC adsorbent to increase the service life of VOC filters. In particular, with photoactive TiO_2_ forms on zeolite, efficient and repetitive photo-regeneration is attainable through the dissociation of VOC molecules by the photocatalytic reaction. We optimized the TiO_2_ coating amount to obtain TiO_2_/zeolite particles with a high surface area (BET surface area > 500 m^2^/g) and high adsorption capacity. A VOC filter with an adsorption efficiency of 72.1% for formaldehyde was realized using TiO_2_/zeolite as the adsorbent. Furthermore, the TiO_2_/zeolite filter exhibits a photo-regeneration efficiency of >90% for the initial two regeneration cycles using ultraviolet illumination, and >60% up to five cycles. Based on these observations, we consider that TiO_2_/zeolite is a potential adsorbent candidate for photo-regenerative VOC filters.

## 1. Introduction

Recently, indoor air filtration received significant attention owing to the growing threat to the environment and human health caused by small-size air pollutants such as volatile organic compound (VOC) gases [1,2,3,4,5]. However, due to the limited adsorption capacity of VOC adsorbents, including activated carbon, a fast breakthrough can occur, which reduces the service life of the filters [6]. For example, in the case of activated carbon fiber (ACF) filters, full saturation and breakthrough can occur within several hours in a 50–500 ppm toluene environment [7]. Therefore, a new strategy that substantially extends the service life of VOC filters is required; in this regard, the regeneration of VOC filters is a feasible strategy. In particular, through the regeneration process, the adsorbed VOC molecules on the filter media can be desorbed or dissociated, thereby recovering the initial filter performance. Thus, the service life can be extended without increasing adsorption capacity. Various adsorbents and regeneration processes were demonstrated previously. For example, Sidheswaran et al. reported on ACF-based VOC filters regenerated via a thermal desorption process [8]. By heating the filter at approximately 150 °C to provide sufficient energy to remove the VOC molecules from the filter, 70–80% regeneration efficiency was achieved. Lv et al. demonstrated a microwave-based regeneration process using hydrophobically modified NaY zeolite filters [9], wherein microwave irradiation induced heating of water molecules, which desorbed toluene molecules and restored the adsorption capacity. Further, Coss et al. reported microwave-regenerated activated carbon filters [10], in which solvent molecules, such as those of tetrachloroethylene, were desorbed from the inner pores of the active carbon via microwave treatment, recovering the surface area and adsorption capacity. Next, Liu et al. reported hybrid TiO_2_/zeolite composite synthesized for H_2_S removal and SO_2_ capture [11]. Here, regeneration was achieved by washing and calcination. Although these previous approaches for regenerative VOC filters are significant, the thermal regeneration process may cause serious issues, such as overheating of the filter system and large energy consumption, hindering the implementation of these approaches in indoor air filtration. Additionally, the microwave-based regeneration process requires a sophisticated microwave generation unit embedded in the filter system. From this perspective, a simple regeneration process is desired for indoor air filtration to eliminate the possible causes of overheating, and reduce energy consumption and system complexity.

Among the various approaches for regeneration of VOC filters, light-induced photocatalytic regeneration (photo-regeneration) is promising, because the process is simple and does not require complex equipment. The VOC molecules adsorbed on the filter media can be readily desorbed or dissociated by light irradiation, owing to the photocatalytic reaction. Therefore, the use of a heater or a microwave can be excluded. In addition, selecting an appropriate adsorbent that can induce photocatalytic reactions is essential for achieving a high regeneration efficiency. Among the various adsorbents, zeolites have high adsorption efficiency for VOC gases due to their nanoporous structure and charge-compensating cations, which promote the adsorption of polar molecules [11]. However, zeolites are mainly composed of insulating oxides such as Al_2_O_3_ and SiO_2_ [12]. Therefore, light-induced photocatalytic reactions are significantly limited. To circumvent this limitation and realize sufficient photo-regeneration using zeolites, a combination with photocatalytic materials such as TiO_2_ is essential [13]. Furthermore, since TiO_2_ can be activated by irradiating ultraviolet (UV) or near-UV visible light, photo-regeneration can be enabled using the TiO_2_/zeolite composite as the adsorbent. For instance, Ichiura et al. prepared a TiO_2_–zeolite sheet by applying a papermaking technique to perform the effective removal of toluene and formaldehyde [14]. Also, Takeuchi et al. suggested TiO_2_/Y-zeolite hybrid photocatalysts by simple impregnation to remove toluene and benzene [15]. Hydrophobic USY zeolite enabled the smooth transfer of aromatic compounds to TiO2 surfaces, which resulted in high adsorption efficiency. Meanwhile, acid leaching was applied to perform high adsorption and photoactivities of TiO_2_/zeolite composite, according to Zhang et al.’s research [16]. The dealumination and decalcification processes during the acid leaching created more micropores, enhancing the degradation rate of RhB, MO, phenol, and HCHO [16].

To realize photo-regenerative VOC filters, we used TiO_2_-coated zeolite (TiO_2_/zeolite) as the filter medium. TiO_2_ was directly formed on the zeolite particles using a sol–gel process, which allowed uniform and conformal coating of the TiO_2_ layer on the zeolites. We optimized the TiO_2_-to-zeolite (T:Z) ratio in the TiO_2_ coating process to obtain a high surface area and adsorption capacity. UV-regenerative VOC filters were then fabricated on corrugated paper substrates using TiO_2_/zeolite via spray-coating. The TiO_2_/zeolite VOC filters exhibit an adsorption efficiency of 72.1% for formaldehyde; the regeneration efficiency is above 90% for up to two regeneration cycles, and above 60% for five regeneration cycles. These results indicate that TiO_2_/zeolite is a promising candidate for use as a VOC adsorbent in photo-regenerative air filtration systems.

## 2. Materials and Methods

TiO_2_ coating on zeolite particles was carried out using a sol–gel method. Initially, 7.5 g of zeolite powder (Zeolite 13X, Jishim Tech, Cheongwon, Chungbuk, South Korea) was added to a mixed solution of ethanol (396 mL) and deionized (DI) water (4 mL). The solution was then stirred at 750 rpm for 30 min to form a zeolite suspension. For the TiO_2_ precursor solution, 2.5, 5, 10, or 15 g of titanium (IV) isopropoxide (97% gravimetric, Sigma-Aldrich, St. Louis, MO, United States) was dissolved in 100 mL of ethanol, and mixed using a vortex mixer. The TiO_2_ precursor solution was then added dropwise to the zeolite suspension. The mixed solution of the zeolite suspension and the TiO_2_ precursor was stirred at 750 rpm for 30 min, and aged for 3 h to form precipitates. The mass ratio of the TiO_2_ (T) precursor solution and zeolite (Z) suspension solution varied in the range 0.5:1–2:1. Subsequently, to remove the excess TiO_2_ precursor that was not coated on the zeolite, centrifugation was performed at 6000 rpm for 10 min. Finally, the collected particles were dried in an oven at 80 °C for 12 h to remove the residual solvent and calcined at 350 °C for 3 h on a hot plate. To investigate the surface morphology and specific surface area of the TiO_2_/zeolite particles, field-emission scanning electron microscopy (FESEM; JSM-7600F, JEOL, Akishima, Tokyo, Japan) and the Brunauer–Emmett–Teller (BET) method (ASAP 2460, Micromeritics, Norcross, GA, USA) were used, respectively. The atomic composition of TiO_2_/zeolite was analyzed using energy-dispersive X-ray spectroscopy (EDS).

VOC filters were fabricated using the synthesized TiO_2_/zeolite particles. A 5 cm × 5 cm corrugate-shaped paper was used as the base substrate. To prepare the solution for spray-coating, DI water and styrene butadiene rubber were used as the solvent and binder, respectively. After a brief stirring of DI water and the binder, the prepared TiO_2_/zeolite particles were added to the solution. The TiO_2_/zeolite particles, binder, and solvent mixture had a mass ratio of 10:1:30. A reference solution was fabricated using bare zeolite particles without TiO_2_. Next, using spray-coating, the prepared solutions were coated on corrugated paper substrates. Finally, the coated samples were dried at 150 °C for 15 min in an oven. The coating and drying processes were repeated three times to achieve an adequate thickness of the zeolite or TiO_2_/zeolite particles on the paper substrate.

The adsorption efficiencies of formaldehyde and toluene were analyzed using a closed test chamber with a volume of ~0.19 m^3^. First, the fabricated VOC filter was placed inside the chamber, where a fan was installed to circulate air. The formaldehyde or toluene solution was evaporated by placing the solution on a hot plate (80 °C). The initial concentrations of formaldehyde and toluene were measured using a gas detection tube (Gastec detector tube, Gastech, Wangara, WA, Australia). Then, the fan was operated to initiate the air circulation and adsorption of VOC gases. The gas concentration was measured periodically to evaluate the concentration change, and the adsorption efficiency was calculated. For the photo-regeneration of VOC filters, the tested VOC filters were exposed to additional formaldehyde or toluene until the samples became saturated. Then, UV light with a peak emission wavelength of ~253 nm was irradiated onto the filter using a UV lamp (UVT-8CC, Boteck, Gunpo, Gyeonggi, South Korea) for 3 h. After regeneration, adsorption and regeneration efficiencies were evaluated.

## 3. Results

### 3.1. Fabrication and Characterization of TiO_2_/Zeolite Particles

**Figure 1:**
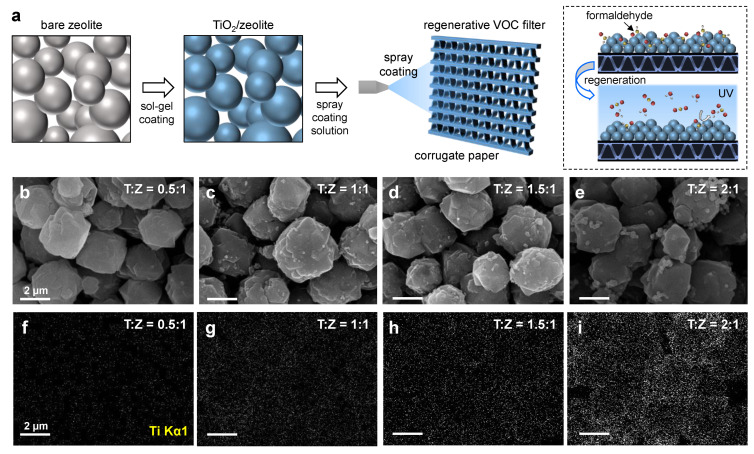
(**a**) Fabrication process and schematic photo-regeneration process of TiO_2_/zeolite-based VOC filters. FESEM images of TiO_2_/zeolite particles fabricated with different T:Z ratios, (**b**) T:Z = 0.5:1, (**c**) 1:1, (**d**) 1.5:1, and (**e**) 2:1. Corresponding EDS elemental mapping images (Ti Ka1) of TiO_2_/zeolite particles fabricated with different T:Z ratios, (**f**) T:Z = 0.5:1, (**g**) 1:1, (**h**) 1.5:1, and (**i**) 2:1. The scale bars in the images indicate 2 μm.

Figure 1a shows the fabrication procedure and schematic photo-regeneration process for TiO_2_/zeolite-based VOC filters. The TiO_2_ layer was coated on bare zeolite particles using a sol–gel process. Then, the fabricated TiO_2_/zeolite particles were dispersed in DI water and spray-coated on a corrugated paper substrate. As described, the TiO_2_ layer plays a crucial role in the photo-regeneration process. However, the surface structure of zeolite can be altered during the TiO_2_ coating process, which may degrade its adsorption properties [17]. Therefore, we first investigated the influence of TiO_2_ coating on the surface morphology, surface area, and pore structure of the zeolites. Figure 1b–e show FESEM images of TiO_2_/zeolite particles synthesized with different TiO_2_-to-zeolite (T:Z) ratios. Here, the T:Z ratio represents the mass ratio of the TiO_2_ (T) precursor solution to the zeolite (Z) suspension solution used for the synthesis, which varies in the range of 0.5:1–2:1. As shown in the FESEM images, the zeolite surface becomes rougher as the T:Z ratio increases. In particular, when the T:Z ratio is 0.5:1, the surface is relatively smooth, without large precipitates (Figure 1b). EDS elemental mapping for Ti Ka1 (Figure 1f–i) suggests that a relatively small amount of TiO_2_ is coated on the surface. By increasing the T:Z ratio, the amount of TiO_2_ coated on the surface increases, which is confirmed by the EDS analysis, and the surface of the zeolite becomes rougher, owing to the precipitates formed on the surface. The size of the precipitates is a few hundred nanometers. Furthermore, the number of precipitates tends to increase with the T:Z ratio, and at T:Z = 2:1, the partial agglomeration of TiO_2_ particles is observed, as shown in Figure 1e.

**Figure 2:**
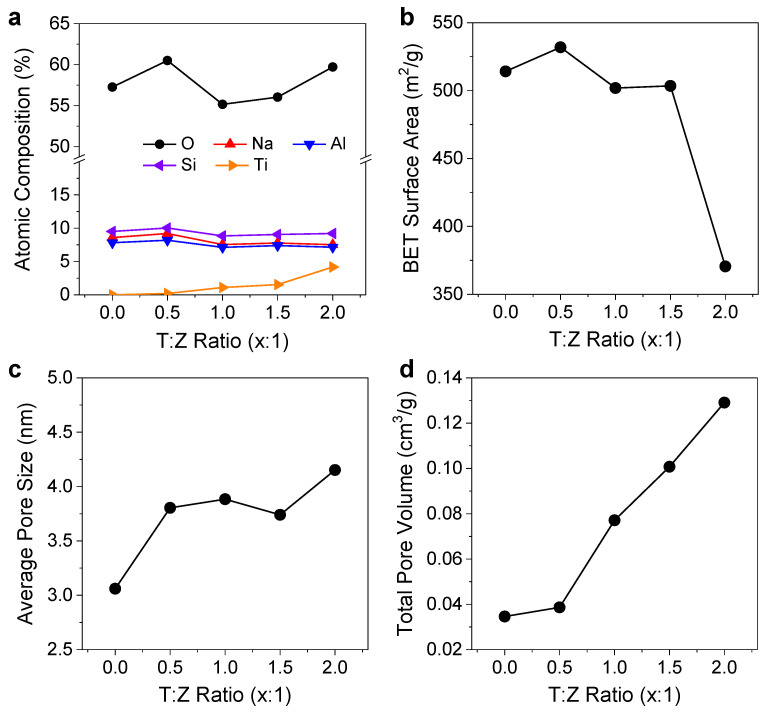
(**a**) Variation in atomic composition of zeolite and TiO_2_/zeolite particle surfaces synthesized with different T:Z ratios (elements: O, Na, Al, Si, and Ti). T:Z ratio of 0:1 indicates bare zeolite particles. Variations in (**b**) BET surface area, (**c**) average pore size, and (**d**) total pore volume of TiO_2_/zeolite particles as a function of T:Z ratio.

**Figure 3:**
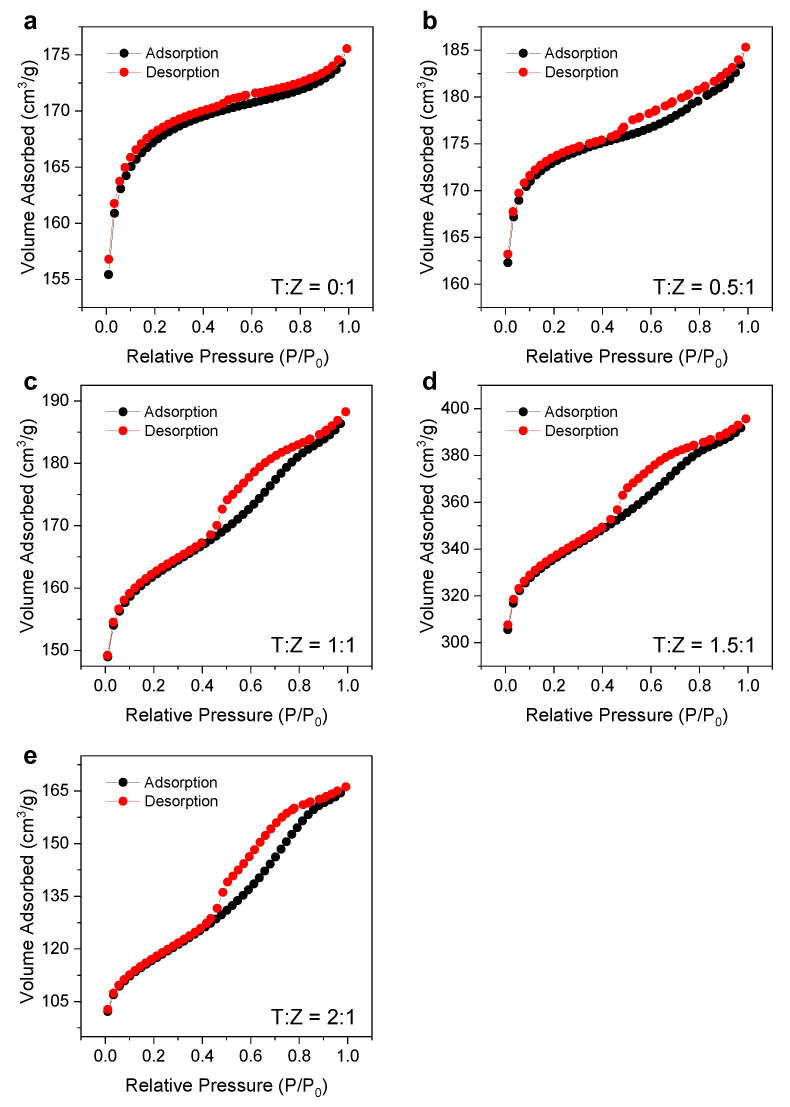
BET nitrogen adsorption and desorption isotherm data of zeolite and TiO_2_/zeolite particles fabricated with different T:Z ratios, (**a**) T:Z = 0:1, (**b**) 0.5:1, (**c**) 1:1, (**d**) 1.5:1, and (**e**) 2:1.

The amount of TiO_2_ coating based on the T:Z ratio was investigated using atomic composition analysis. Figure 2a and Table 1 show the atomic composition ratios of zeolite and TiO_2_/zeolite particle surfaces as a function of T:Z ratio. Ti shows an increasing tendency with the T:Z ratio. For instance, when the T:Z ratio changes from 0.5:1 to 2:1, the Ti composition ratio increases from 0.21% to 4.18%. This indicates that the amount of TiO_2_ formed on the zeolite can be controlled by the T:Z ratio, which affects the degree of photocatalytic reaction under UV illumination. Despite this, we examined the variation in the specific surface area, total pore volume, and pore size of TiO_2_/zeolite particles depending on the T:Z ratio, since the formation of TiO_2_ can alter the surface structures of zeolite. Figure 2b shows the BET surface areas of the bare zeolite (T:Z = 0:1) and TiO_2_/zeolite fabricated with different T:Z ratios. Corresponding nitrogen adsorption and desorption isotherm data are shown in Figure 3a–e. The BET surface area of the TiO_2_/zeolite particles shows a relatively small variation when the T:Z ratio is in the range of 0.5:1–1.5:1, maintaining a value over 500 m^2^/g, which is similar to that of bare zeolite (514 m^2^/g). However, when the T:Z ratio is further increased to 2:1, the BET surface area decreases to 370 m^2^/g, implying that the surface structure of the zeolite is significantly altered. Furthermore, the average pore size shows a significant increase from ~3.06 nm to ~4.15 nm when the T:Z ratio is increased to 2:1 (Figure 2c). This indicates that the surface structure is modified at this ratio, which can be attributed to the overloading of the TiO_2_ precursor. Further, the total pore volume shows a monotonous increase with the T:Z ratio, as shown in Figure 2d. Therefore, considering the efficiency of VOC adsorption and the regeneration capability, we consider 1.5:1 as the optimal T:Z ratio for regeneratable VOC filters.

### 3.2. Fabrication of TiO_2_/Zeolite-Based VOC Filters

**Figure 4:**
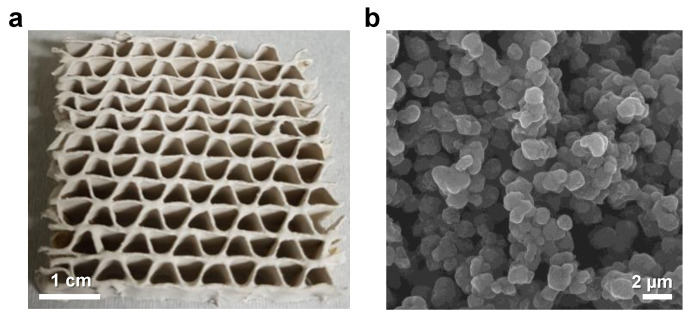
(**a**) Optical images of TiO_2_/zeolite VOC filter fabricated using the spray-coating method. (**b**) FESEM images of TiO_2_/zeolite particles coated on the VOC filter.

Using the optimized TiO_2_/zeolite particles (T:Z ratio of 1.5:1), VOC filters were fabricated using the spray-coating method. Here, corrugated paper was used as the filter substrate. Owing to the web-like flute structure of the corrugated paper, a relatively large area for VOC adsorption can be provided, allowing a higher adsorption capacity compared to flat substrates. Figure 4a shows the optical images of the fabricated TiO_2_/zeolite-based VOC filter. TiO_2_/zeolite particles were uniformly coated on the corrugated paper, including the inner parts of the core flute, owing to the advantage of the spray-coating method. Figure 4b shows the FESEM image of the filter surface. The TiO_2_/zeolite formed on the corrugated paper has an open stacking structure. This enabled easy penetration of VOC gases into the bottom parts of the TiO_2_/zeolite layer, and enlarged the adsorption area. Furthermore, concerning the mechanism of photocatalytic regeneration process, the following photocatalytic reaction can take place. Initially, when the TiO_2_/zeolite filter is exposed to VOC gases, including formaldehyde (HCHO), VOC molecules are adsorbed physically or chemically on the surface depending on the molecular structure of the VOC gas [18,19]. For instance, in the case of formaldehyde, the molecules adsorbed on the TiO2 surface can be explained with monodentate and bidentate adsorption configurations [18]. Generally, a monodentate configuration depicts the O atom of HCHO (O_F_) bound to the surface under-coordinated Ti atom. For the five-fold Ti_5_ atom, binding with the O_F_ atom shows the adsorption energy of 0.472 eV and the bond length of 2.271 Å. For the four-fold Ti_4_ atom, adsorption energy of 0.907 eV and bond length of 2.168 Å are shown [18]. Based on the Bader charge analysis, part of the electron diffuses during the interaction between HCHO molecules and Ti atom surface. This process results in the weakening of the π bond and the elongation of C=O_F_. Further, chemical changes in the O and C atoms of formaldehyde differ with coverages [19]. At low coverages below the 0.25 monolayer, as in the case of VOC gases, the HCHO molecule binds to surface 5-coordinated Ti atoms via the O atom. However, after continuous exposure to formaldehyde, adsorption saturates, and a breakthrough occurs; thus, the VOC filtration ability is lost. UV illumination was applied to the filter to regenerate it and recover its adsorption capability. Under UV irradiation, TiO_2_ induces the oxidation of formaldehyde molecules via the following reactions [20]:

(1) $$\mathrm{HCHO}+H_{2}O+2h^{+}\to \mathrm{HCOOH}+2H^{+}$$

(2) $$\mathrm{HCOOH}+2h^{+}\to {\mathrm{CO}}_{2}+2H^{+}$$

dissociating the adsorbed formaldehyde molecules. In particular, under UV irradiation, electron–hole (e–h) pairs are generated in the TiO_2_ layer. The photogenerated holes then contribute to the conversion of formaldehyde into formic acid (HCOOH), and finally to CO_2_ [20]. In addition, partial oxidation byproducts such as HCOOH are oxidized to CO_2_ with sufficient UV illumination.

### 3.3. Adsorption Efficiency and Regeneration Efficiency for Formaldehyde

**Figure 5:**
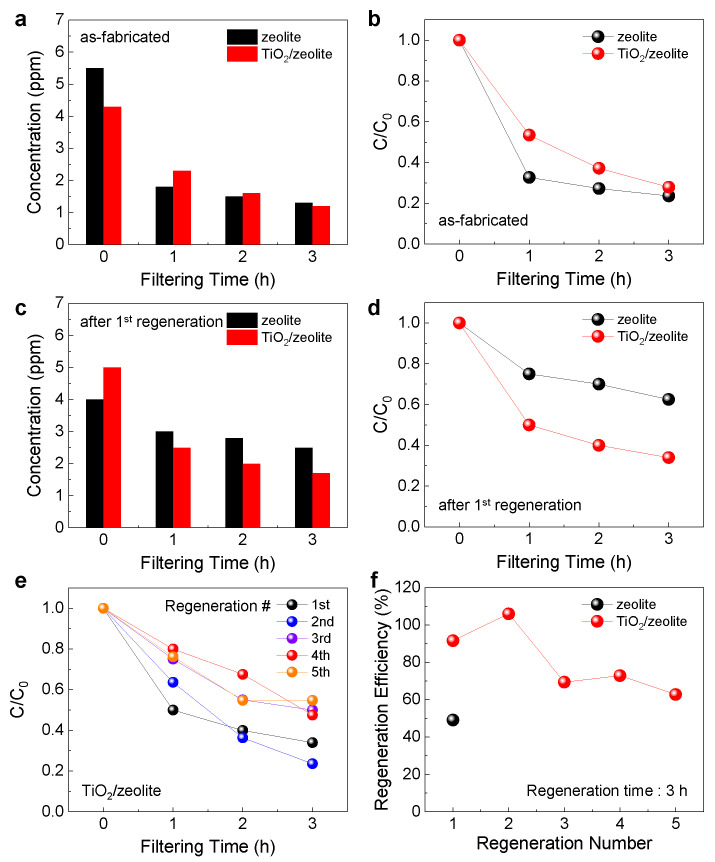
(**a**) Variation in formaldehyde concentration for bare zeolite and TiO_2_/zeolite VOC filters as a function of filtering time. (**b**) Relative formaldehyde concentration (C/C_0_) changes as a function of filtering time. Here, C_0_ and C indicate formaldehyde concentration before and during the filtering process, respectively. (**c**) The variation in formaldehyde concentration and (**d**) C/C_0_ as a function of filtering time after the first regeneration process. (**e**) Relative formaldehyde concentration (C/C_0_) change for five regeneration processes (TiO_2_/zeolite filter). (**f**) Variation in regeneration efficiency as a function of regeneration number.

For the TiO_2_/zeolite VOC filter, the adsorption efficiency for formaldehyde was evaluated. To analyze the adsorption efficiency, the filter was placed inside a chamber, and the change in formaldehyde concentration (C) was measured every 1 h for up to 3 h. Figure 5a shows the variation in formaldehyde concentration as a function of filtering time for bare zeolite- and TiO_2_/zeolite-based VOC filters. Figure 5b shows the relative concentration (C/C_0_) change as a function of the filtering time, where C_0_ denotes the initial concentration of formaldehyde before filtering. For both filters, the concentration of formaldehyde decreases as the filtering time increases, indicating the effective capture of formaldehyde by the zeolite-based filters. After 3 h of filtering, the C/C_0_ values decrease to 0.236 and 0.279 for the zeolite and TiO_2_/zeolite VOC filters, respectively. The slightly higher C/C_0_ value for the TiO_2_/zeolite VOC filter can be attributed to the decrease in the BET surface area after TiO_2_ coating. To evaluate the regeneration efficiency, both the zeolite and TiO_2_/zeolite VOC filters were first saturated with formaldehyde gas by placing the filters in a formaldehyde-rich environment. Next, UV exposure was performed for 3 h, and the filtering tests were repeated. Figure 5c,d show the formaldehyde concentration and C/C_0_ as functions of the filtering time after the first regeneration. The decrease in C/C_0_ for the bare zeolite filter is significantly reduced, indicating degradation of the adsorption efficiency. In contrast, in the case of the TiO_2_/zeolite VOC filter, the decrease in C/C_0_ is similar to that before regeneration. To evaluate the repetitive regeneration of TiO_2_/zeolite VOC filters, the regeneration process was performed for five cycles. Figure 5e,f show the C/C_0_ and regeneration efficiencies, respectively, for five regeneration cycles. Regeneration efficiency is defined as the following equation:

(3) $$\text{Regeneration efficiency}(\%)=\frac{\text{adsorption efficiency after regeneration}}{\text{Initial adsorption efficiency}}$$

In the absence of TiO_2_ (bare zeolite filter), the regeneration efficiency after the first regeneration cycle is below 50% (Figure 5f). This indicates that UV irradiation may have limited effects on dissociating or desorbing the formaldehyde molecules adsorbed on the zeolite surface. However, with TiO_2_, the adsorption efficiency is restored close to its initial value, particularly in the first two regeneration processes, indicating the efficient removal of formaldehyde by the photocatalytic reaction. The regeneration efficiency is over 90% during the first two regeneration cycles, and decreases to approximately 60% afterwards. The decrease in regeneration efficiency after repeated cycles is attributed to non-uniform UV irradiation on the VOC filter owing to the corrugated structure of the paper substrate. Nevertheless, the results show that TiO_2_ on zeolite has a significant effect on enhancing the regeneration efficiency of zeolite-based VOC filters.

### 3.4. Adsorption Efficiency and Regeneration Efficiency for Toluene

**Figure 6:**
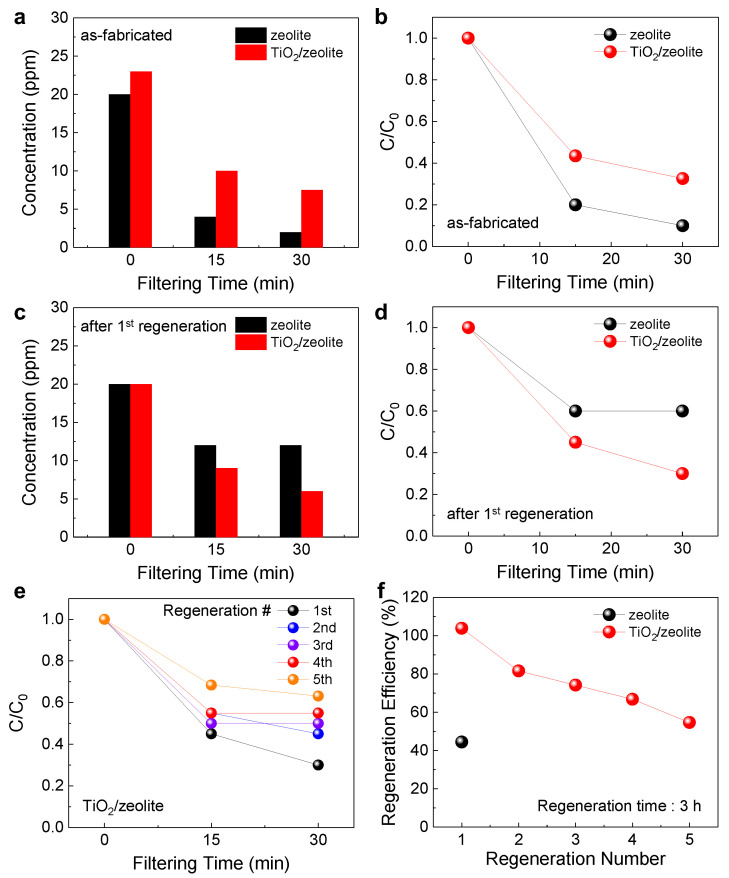
(**a**) Variation in toluene concentration for bare zeolite and TiO_2_/zeolite VOC filters as a function of filtering time. (**b**) Relative toluene concentration (C/C_0_) changes as a function of filtering time. Here, C_0_ and C indicate toluene concentration before and during the filtering process, respectively. (**c**) Variation in toluene concentration and (**d**) C/C_0_ as a function of filtering time after the first regeneration process. (**e**) Relative toluene concentration (C/C_0_) change for five regeneration processes (TiO_2_/zeolite filter). (**f**) Variation in regeneration efficiency as a function of regeneration number.

In addition to formaldehyde, the toluene adsorption and regeneration efficiencies were investigated. Similarly, adsorption efficiency was measured using a closed test chamber. However, in this case, the change in toluene concentration was measured every 15 min for up to 30 min, because of the relatively rapid adsorption of toluene molecules. Figure 6a,b show the toluene concentration change and relative concentration (C/C_0_) variation for the bare zeolite and TiO_2_/zeolite VOC filters, respectively, as a function of filtering time. Similar to formaldehyde, the bare zeolite filter exhibits a relatively higher adsorption efficiency before regeneration. However, after regeneration by UV exposure for 3 h, the TiO_2_/zeolite VOC filter shows higher adsorption efficiency, as shown in Figure 6c,d. In particular, the bare zeolite filter has a limited adsorption capability, with the C/C_0_ value saturated at approximately 0.6. In contrast, the adsorption efficiency of the TiO_2_/zeolite filter is close to that of the as-fabricated filter. The repetitive regeneration performance for toluene was also evaluated. Figure 6e,f show the C/C_0_ and corresponding regeneration efficiencies of the bare zeolite and TiO_2_/zeolite filters for five regeneration cycles, respectively. Similar to that of formaldehyde, the regeneration efficiency is significantly lower for the bare zeolite filter, owing to the absence of the TiO_2_ photocatalytic layer. In particular, after the first regeneration, the regeneration efficiency of the bare zeolite filter is 44.4%, whereas in the case of TiO_2_/zeolite filters, the regeneration efficiency is above 80% during the initial two cycles, and above 50% up to five cycles. Although further improvements in achieving a high regeneration efficiency are still required, for example, through the optimization of the regeneration process, the implementation of the TiO_2_ photocatalytic layer has a substantial influence on improving the regeneration efficiency for various VOC gases. Also, it is of note that the regeneration efficiency obtained by photo-regeneration is comparable to that using thermal- or microwave-assisted regeneration processes, indicating that the synergetic combination of TiO_2_/zeolite adsorbent and photo-regeneration is promising for extending the service life of VOC filters.

## 4. Conclusions

In this study, we demonstrate UV-regenerative VOC filters using TiO_2_/zeolite adsorbents. TiO_2_/zeolite particles with surface properties similar to those of bare zeolite are achieved by employing a solution-based coating method and an optimized synthesis protocol. Furthermore, using the spray-coating method, VOC filters were fabricated on a corrugated paper membrane. From the VOC adsorption and UV-mediated regeneration tests for formaldehyde and toluene, the TiO_2_/zeolite filters show ameliorated regeneration efficiency compared to the bare zeolite-based filters. Based on these results, we propose that the TiO_2_/zeolite VOC filter is a potential adsorbent candidate for multi-use UV-regenerative air filtration systems.

## Figures and Tables

**Table 1:** Atomic compositions of zeolite and TiO_2_/zeolite particle surfaces synthesized with different T:Z ratios (in %).

T:Z Ratio	O	Na	Al	Si	Ti
0:1	57.27	8.59	7.82	9.52	0
0.5:1	60.50	9.21	8.20	10.03	0.21
1:1	55.16	7.55	7.13	8.85	1.10
1.5:1	56.04	7.78	7.39	9.06	1.54
2:1	59.71	7.52	7.15	9.20	4.18

## Data Availability

Not applicable.
